# Functional Analysis of GRSF1 in the Nuclear Export and Translation of Influenza A Virus mRNAs

**DOI:** 10.3390/v16071136

**Published:** 2024-07-16

**Authors:** Jordana Schmierer, Toru Takimoto

**Affiliations:** Department of Microbiology and Immunology, University of Rochester Medical Center, Rochester, NY 14642, USA; jordana_schmierer@urmc.rochester.edu

**Keywords:** influenza A virus, GRSF1, mRNA nuclear export, translation, host adaptation

## Abstract

Influenza A viruses (IAV) utilize host proteins throughout their life cycle to infect and replicate in their hosts. We previously showed that host adaptive mutations in avian IAV PA help recruit host protein G-Rich RNA Sequence Binding Factor 1 (GRSF1) to the nucleoprotein (NP) 5’ untranslated region (UTR), leading to the enhanced nuclear export and translation of NP mRNA. In this study, we evaluated the impact of GRSF1 in the viral life cycle. We rescued and characterized a 2009 pH1N1 virus with a mutated GRSF1 binding site in the 5’ UTR of NP mRNA. Mutant viral growth was attenuated relative to pH1N1 wild-type (WT) in mammalian cells. We observed a specific reduction in the NP protein production and cytosolic accumulation of NP mRNAs, indicating a critical role of GRSF1 in the nuclear export of IAV NP mRNAs. Further, in vitro-transcribed mutated NP mRNA was translated less efficiently than WT NP mRNA in transfected cells. Together, these findings show that GRSF1 binding is important for both mRNA nuclear export and translation and affects overall IAV growth. Enhanced association of GRSF1 to NP mRNA by PA mutations leads to rapid virus growth, which could be a key process of mammalian host adaptation of IAV.

## 1. Introduction

Influenza A virus (IAV) is among the most common causes of human respiratory infections and poses an enormous and unpredictable global public health threat [1]. Influenza pandemics occur when a new strain crosses the species barrier and acquires the ability to replicate and transmit in human hosts [2]. In general, avian IAVs do not infect human cells well, mainly due to receptor specificity and inefficient genome replication. It is widely accepted that the avian IAV polymerase requires mutations, such as PB2 627K, to function well in mammalian hosts [3]. In 2009, a new pandemic H1N1 (pH1N1) virus emerged and rapidly spread worldwide, causing a global pandemic. Unlike previous pandemics, the pH1N1 virus is a swine-origin strain with a unique genomic constellation derived from avian-, swine-, and human-origin viral genes. Two of the three polymerase genes, PA and PB2, are derived from avian IAV, which is of note given that avian IAV polymerases do not function well in mammalian cells. Importantly, pH1N1 PB2 does not have the human adaptive PB2 627K mutation, which is required for species-specific interaction with acidic nuclear phosphoprotein 32 (ANP32) to form polymerase oligomers and replicate the viral genome [4]. We have been studying mutations within the PA and PB2 genes of pH1N1 responsible for mammalian host adaptation [5,6,7,8,9]. Although some mutations in pH1N1 PB2 partially contribute to enhanced polymerase activity, we found that PA was the most significant component of enhanced pH1N1 polymerase activity. An avian polymerase complex whose PA was replaced with that of pH1N1 was fully functional and replicates the viral genome in mammalian cells, suggesting that mutations in PA can activate the avian viral polymerase without the host adaptive PB2 627K mutation [6].

Previously, we identified key residues responsible for mammalian host adaptation in both the PA N-terminal domain (NTD) and C-terminal domain (CTD) [6,8,9]. Further analysis revealed that PA mutations in the NTD enhance the association of host RNA binding protein G-rich RNA sequence binding factor 1 (GRSF1) to viral nucleoprotein (NP) mRNA at the specific binding signal in the 5’ untranslated region (UTR). GRSF1 binding to NP mRNA accelerated its nuclear export and translation efficiency [9]. GRSF1 is a member of the heterogeneous nuclear ribonucleoprotein (hnRNP) F/H family and is known to bind cellular mRNAs such as *Use1* and *GPx4* through either G-rich sequences in the 5’ UTR or through formation of guanine quadruplex (G4) structures in the 5’ UTR [10,11,12]. Previous studies have shown that GRSF1 binds the sequence ^10^AGGGU^14^ in the 5’ UTR of IAV nucleoprotein (NP) and non-structural protein (NS) mRNAs [13,14]. In contrast, the 5’ UTR of IAV PB1 mRNAs contains the sequence ^10^AGGCA^14^ and was shown to not interact with GRSF1 [14]. Using a reporter gene containing the IAV NP 5’ UTR, we generated a mutation at the GRSF1 binding site from AGGGU (wild-type, WT) to AGGCA (CA mutant). We found significantly less CA mutant reporter mRNA bound to GRSF1 relative to the wild-type (WT) reporter mRNA in cells expressing pH1N1 polymerase complex [9]. We also observed reduced nuclear export and protein expression of the CA reporter, indicating that GRSF1 plays a role in the translation efficiency of IAV NP mRNA.

Of the eight IAV gene segments, NP and NS1 are expressed at the highest levels [15,16]. NP is required to encapsulate the viral genome as it is being replicated to stabilize the progeny genome and to prevent recognition of naked RNA by innate immune sensors [17,18]. NS1 antagonizes the innate immune system by blocking host mRNA processing and preventing innate immune signaling pathways [19,20,21]. Both proteins serve important functions in the progression of the viral life cycle, and regulation of their protein synthesis is essential for efficient genome replication and a productive infection. Therefore, enhanced association of GRSF1 to specifically increase the translation efficiency of NP and NS1 could be a major strategy of IAV host adaptation. However, the actual impact of GRSF1 association on virus infection, replication, and spread has not been directly evaluated. Here, we address the role of GRSF1 in NP protein synthesis during the viral life cycle using a 2009 pH1N1 virus containing a mutation at the GRSF1 binding site in the NP gene. We observed reduced nuclear export of NP mRNA, NP expression, genome replication, and virus growth. Together, these results indicate the strong impact of GRSF1 in the life cycle of IAV.

## 2. Materials and Methods

### 2.1. Cell Lines and Viruses

MDCK (ATCC: CCL-34), A549 (ATCC: CRM-CCL-185), and 293T (ATCC: CRL-3216) cells were maintained in Dulbecco’s Modified Eagle’s Medium (DMEM, Gibco, Grand Island, NY, USA) supplemented with 8% FB Essence (Avantor, Center Valley, PA, USA), 25 mM HEPES (Gibco), and 50 μg/mL gentamicin (Gibco). Wild-type and recombinant A/California/04/2009 (H1N1) (Cal) with the GRSF1 CA binding mutation (^10^AGGGU^14^ mutated to ^10^AGGCA^14^) in the NP mRNA 5’ UTR were rescued in 293 T/MDCK cell co-culture [6,9,22]. Rescued viruses were sequenced for confirmation. Viral infections were carried out in DMEM containing 0.15% bovine serum albumin (DMEM-BSA), 25 mM HEPES (Gibco), and 50 μg/mL gentamicin (Gibco), with or without acetylated trypsin at 2 μg/mL.

### 2.2. Viral Infections

For a single-cycle virus growth, A549 cells were infected with Cal WT or Cal NPCA viruses at an MOI of 0.2 for 1 h at 37 °C. At specific time points post infection, supernatants were collected and treated with acetylated trypsin at 2 μg/mL for 1 h. The virus was titrated by measuring TCID_50_ in MDCK cells.

For monitoring a multi-cycle viral growth, MDCK cells were infected with viruses at an MOI of 0.01 for 1 h at 37 °C and cultured in DMEM-BSA supplemented with acetylated trypsin at 2 μg/mL. At indicated time points, supernatants were collected and titrated by measuring TCID_50_ in MDCK cells.

For protein expression analysis, A549 cells were infected with Cal WT or Cal NPCA viruses at an MOI of 2 for 1 h at 37 °C. At indicated time points, cells were lysed with RIPA lysis buffer containing Halt protease inhibitor (PI87785, ThermoFisher, Waltham, MI, USA) and cleared samples were analyzed by Western blotting.

### 2.3. Western Blotting and Reagents

For immunoblotting, cell lysates were mixed with 4X NuPAGE LDS sample buffer (NP0007, ThermoFisher) and 5% beta-mercaptoethanol. Lysates were resolved by SDS-PAGE and transferred to 0.45 μM PVDF membranes. Primary antibodies used for the detection were mouse anti-PA (1:500 dilution, F5-32 [23]), mouse anti-PB1 (1:500 dilution, F5-10 [23]), rabbit anti-NP (1:3000 dilution, 125989, GeneTex, Irvine, CA, USA), mouse anti-beta actin (1:6000 dilution, 8H10D10, Cell Signaling Technology, Danvers, MA, USA), mouse anti-M1 (1:3000, 76107, GeneTex), and rabbit anti-GRSF1 (1:3000 dilution, A305-136A, Bethyl Laboratories, Montgomery, TX, USA). Secondary antibodies used were horse anti-mouse IgG HRP-linked (1:3000 dilution, 7076, Cell Signaling Technology), goat anti-rabbit IgG HRP-linked (1:3000 dilution, 7074, Cell Signaling Technology), and goat anti-mouse IgG1 HRP-linked (1:3000 dilution, ab97240, abcam). Samples were visualized using SuperSignal™ West Dura Extended Duration Substrate (ThermoFisher) with a BioRad Chemidoc XRS Imager. Pixel density of the images was measured using ImageJ software version 1.54. Band intensity for independent replicates as indicated for each figure was normalized to that of the corresponding β-actin loading control for each sample. The data of NPCA samples were normalized to NP WT samples. Data are presented as the average pixel intensity of NPCA samples compared to NP WT samples.

### 2.4. RNA Quantification

For measuring viral NP mRNA, cRNA, and vRNA, A549 cells were infected with Cal WT or the Cal NPCA virus at an MOI of 0.2 for 1 h and cultured for 8 h at 37 °C. Infected cells were lysed with TRIzol Reagent (Invitrogen, Waltham, MA, USA) and RNA was extracted according to the manufacturer’s protocol. The RNA concentration and purity was determined with a Nanodrop 2000 (ThermoFisher). An amount of 100 ng of purified RNA was used for RT-PCR with the hot-start modification using RevertAid Reverse Transcription Kit (ThermoFisher). Primers used for RT-PCR and qPCR were designed to be strand specific for all three NP RNA species using tags unrelated to IAV and validated as described by Kawakami et al. [24]. cDNAs were generated from RNA with a primer that annealed to a unique feature of each RNA and contained at the 5’ end an mRNA tag (Cal NP mRNA Tag: CCAGATCGTTCGAGTCGTTTTTTTTTTTTTTTTTCCTCAACTGTC), a cRNA tag (Cal NP cRNA tag: GGATCCTAATACGACTCACTATAGGGGTAGAAACAAGGGTATTT-TTCCT), or a vRNA tag (Cal NP vRNA Tag: GGCCGTCATGGTGGCGAATAAATGGACGAAGGACAAGGGTTGC). Real-time PCR was carried out using SYBR Green PCR Master Mix (Applied Biosystems, Waltham, MA, USA) with an Applied Biosystems QuantStudio 3 Real-time PCR system. qPCR primers were specific to NP mRNA (Cal NP 1466F: CGATCG-TGCCTTCCTTTG and mRNA tag R: CCAGATCGTTCGAGTCG), cRNA (Cal NP 1466F: CGATCGTGCCTTCCTTTG and cRNA tag R: GCTAGCTTCAGCTAGGCATC), or vRNA (Cal NP 846R: CCTCAGAATGAGTGCTGACCGT and vRNA tag F: GGCCGTCA-TGGTGGCGAAT). DNA standards for Cal NP mRNA, Cal NP cRNA, and Cal NP vRNA were generated by PCR. A standard curve from 10^1^ to 10^9^ copies was used for quantification.

### 2.5. Nuclear/Cytosolic Fractionation

A549 cells were infected with either Cal WT or a Cal NPCA virus at an MOI of 3 for 1 h at 37 °C. At 8 hpi, cells were treated with cytoplasmic extraction buffer (10 mM HEPES, 60 mM KCl, 1 mM DTT, 1 mM EDTA, 0.05% NP-40) and centrifuged at 2250× *g* for 10 min at 4 °C. RNAs in the supernatant were isolated by TRIzol Reagent as described above. The cell pellet containing nuclear RNA was washed three times with ice-cold PBS to remove any remaining cytosolic RNA. Nuclear RNA was isolated by TRIzol Reagent as described above. Nuclear and cytosolic RNA were used for qRT-PCR as described above. NP mRNA primers were used as described above. PB1 mRNA primers were designed to add an mRNA tag during cDNA synthesis (Cal PB1 mRNA RT: CCAGATCGTTCGAGTCGTTTTTTTTTTTTTTTTTCATGAAGGA-CAA) and qPCR primers were designed to be specific to PB1 mRNA (Cal PB1 qPCR 2182F: TCTAGGGCCCGGATTGAT and mRNA tag R: CCAGATCGTTCGAGTCG). cDNAs were generated from GAPDH mRNA and U6 snRNA using Random Hexamer Primers (ThermoFisher). qPCR primers were specific to GAPDH (GAPDH F: ACAGTCAGCCGCATCTTCTT and GAPDH R: GTCTTCTGGGTGGCAGTGAT) or U6 (U6 F: CTCGCTTCGGCAGCACAT and U6 R: AACGCTTCACGAATTTGCGT). DNA standards for Cal NP mRNA, Cal PB1 mRNA, GAPDH mRNA, and U6 snRNA were generated by PCR. A standard curve from 10^1^ to 10^9^ copies was used for quantification.

### 2.6. In Vitro Transcription of Capped and Polyadenylated RNAs and Transfection

Cal NP WT and NPCA cDNAs were subcloned from pPolI expression vectors used to rescue the respective viruses into a pTF1 plasmid, which contains the T7 promoter [25]. Capped and polyadenylated RNAs were then transcribed in vitro using the HiScribe T7 ARCA mRNA Kit (with tailing) (E2060S, New England BioLabs, Ipswich, MA, USA) according to the manufacturer’s protocol. Then, 293T cells in a 12-well plate were transfected with 1 μg in vitro-transcribed mRNA. At 4 h post transfection, cells were lysed with RIPA lysis buffer containing Halt protease inhibitor (PI87785, ThermoFisher). Protein expression was measured by Western blot and quantified as described above.

### 2.7. Transfection Based Polyribosome Fractionation

Next, 293T cells in 100 mm dishes were transfected with 16 μg each of in vitro-transcribed NP WT or NPCA mRNA by Lipofectamine 2000 in Opti-MEM for 16 h at 37 °C. Polysome fractionation was carried out as described by Panda et al. [26]. Briefly, cells were washed three times with ice-cold PBS containing 100 μg/mL CHX (ThermoFisher) and then lysed in 750 μL of high-salt lysis buffer (300 mM NaCl, 20 mM Tris-HCl pH 7.5, 10 mM MgCl2, 1x HALT protease inhibitor, 120 units RiboLock RNase Inhibitor (ThermoFisher), and 100 μg/mL CHX). After centrifugation at 15,000× *g* for 10 min, the total RNA concentration in the supernatant was determined by a NanoDrop 2000. A total of 220 μg of RNA from transfected cells was loaded on top of a 10–50% linear sucrose gradient prepared in high-salt lysis buffer and centrifuged for 90 min at 39,000 rpm at 4 °C using a Beckman Coulter SW 41Ti rotor. Gradients were fractionated using a BR-188 Density Gradient Fractionation System (Brandel, Gaithersburg, MD, USA), with absorbance measured at 254 nm. Gradients were separated into 18 fractions. The RNA in each fraction was extracted by TRIzol and precipitated with isopropanol at −20 °C. RT-PCR for NP and GAPDH mRNAs was carried out as described above. The analysis of mRNA distribution amongst polysome fractions was determined as described by Panda et al. [26].

## 3. Results

### 3.1. Effect of Mutation at the GRSF1 Binding Site on Virus Growth

**Figure 1:**
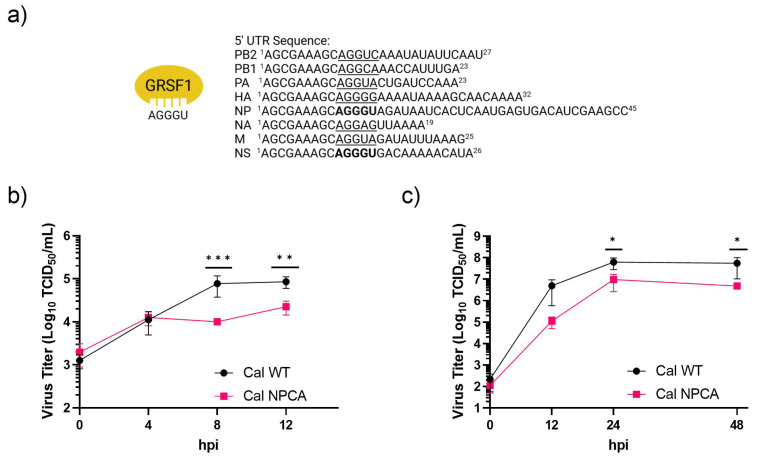
Attenuated NPCA virus growth in cultured cells. (**a**) Schematic of GRSF1 binding site AGGGU and nucleotide identity in pH1N1 Cal WT gene segments. Sequences of 5’UTR of each viral segment are shown as positive sense. Nucleotides at 10–14 of each gene segment are underlined or bolded. (**b**) A single-step growth curve of indicated viruses in A549 cells infected at an MOI of 0.2. At indicated times, virus in the supernatant was collected and titrated. (**c**) MDCK cells were infected with the indicated viruses at an MOI of 0.01 and cultured in the presence of acetylated trypsin. At indicated times, virus in the supernatant was collected and titrated. All error bars show means plus/minus the standard deviations (n  =  3 biological replicates). Two-way ANOVA followed by Tukey’s multiple comparison test in PRISM (* *p*  <  0.05, ** *p*  <  0.01, *** *p*  <  0.001).

To analyze the impact of GRSF1 on virus replication, we rescued a 2009 pH1N1 (Cal) virus containing a mutation at the GRSF1 binding site in the NP gene using a reverse genetics system. GRSF1 is known to bind to the sequence AGGGU, and this motif is found at nucleotides 10–14 in the 5’ UTR of NP and NS mRNAs (Figure 1a). Both proteins play key roles in the viral life cycle and are needed at high quantities early during infection to replicate viral genes efficiently. All other gene segments have different nucleotides at positions 13 and 14, including polymerase protein genes that are expressed at low levels in infected cells [15]. PB1 has the sequence AGGCA, which has been shown to not be recognized by GRSF1 [14]. We mutated nucleotides 13–14 in the Cal NP 5’ UTR to CA. We rescued both Cal WT and a mutant virus Cal NPCA to directly evaluate the role GRSF1 plays in the viral life cycle. First, we measured single-cycle growth of the viruses in human alveolar basal epithelial A549 cells. Compared to Cal WT, the production of progeny Cal NPCA was limited at both 8 h and 12 h post infection (hpi) (Figure 1b). We next measured multi-step growth in MDCK cells in the presence of acetylated trypsin. Similarly, we observed that Cal NPCA grew more slowly and to lower titers relative to Cal WT (Figure 1c). These results suggest that the presence of the GRSF1 binding sequence in the 5’ UTR of NP mRNA contributes to efficient viral growth in cultured cells.

### 3.2. Specific Reduction of NP Protein Expression in Cal NPCA-Infected Cells

**Figure 2:**
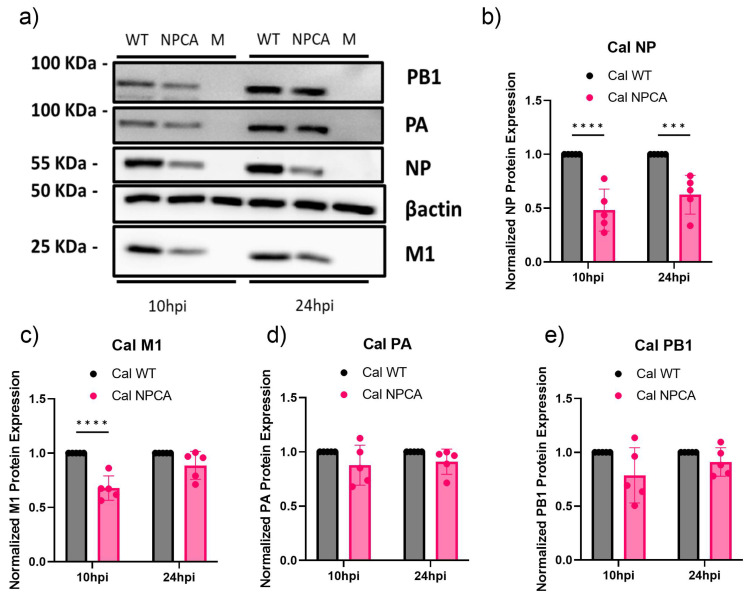
Viral protein expression in infected cells. (**a**) A549 cells were infected at an MOI of 2 and cell lysates were collected at 10 and 24 hpi. Representative image of immunoblot analysis of PB1, PA, NP, M1, and β-actin in cell lysates using specific antibodies. M: mock infection. (**b**–**e**) Relative viral protein expression was calculated by densitometry analyses, normalized to actin, and expressed as each respective Cal NPCA protein expression relative to Cal WT. All error bars show means plus/minus the standard deviations (n  =  5 biological replicates). Two-way ANOVA followed by Tukey’s multiple comparison test in PRISM (*** *p*  <  0.001, **** *p* < 0.0001).

In our previous in vitro studies, we observed reduced protein expression of the reporter mRNA mutated at the GRSF1 binding site [9]. We next tested whether NP protein expression would be similarly reduced in cells infected with Cal NPCA. A549 cells were infected with either Cal WT or Cal NPCA, and cell lysates were collected at 10 and 24 hpi. We measured the viral protein expression of NP and polymerase PB1 and PA proteins, as well as M1 (Figure 2a). Among the viral proteins tested, NP protein expression was most significantly reduced in Cal NPCA-infected cells relative to WT at both 10 and 24 hpi as expected (Figure 2a,b). We also detected a slight reduction in production of other viral proteins (Figure 2c–e), especially M1 (Figure 2c) that is expressed abundantly in infected cells compared to polymerase PA and PB1 proteins (Figure 2d,e) [15,27]. This protein reduction likely reflects attenuated genome replication due to reduced NP production in Cal NPCA-infected cells, as vRNA is a template to further produce mRNAs. The effect of CA mutation on NP protein production was evident even at 24 hpi. Together, these data suggest that the GRSF1 binding site in the 5’ UTR of NP mRNA plays a major role in NP protein expression, which potentially impacts the levels of other proteins’ expression in infected cells.

### 3.3. Impact of CA Mutation on Viral Genome Replication

**Figure 3:**
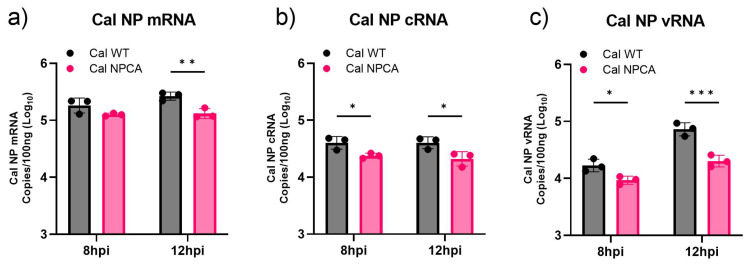
RNA production in infected cells. A549 cells were infected with indicated viruses at an MOI of 0.2 At indicated times, total RNA was extracted, and Cal NP mRNA (**a**), cRNA (**b**), and vRNA (**c**) were quantitated by strand-specific qRT-PCR. All error bars show means plus/minus the standard deviations (n  =  3 biological replicates). Two-way ANOVA followed by Tukey’s multiple comparison test in PRISM (* *p*  <  0.05, ** *p*  <  0.01, *** *p*  <  0.001).

Next, we investigated the effect of the CA mutation on viral genome replication in infected cells. Total RNAs were extracted from infected A549 cells at 8 and 12 hpi, and cRNA, vRNA, and mRNAs of the NP segment were quantitated using strand-specific qRT-PCR [24]. We observed that mRNA levels were not significantly reduced in the Cal NPCA virus at 8 hpi (Figure 3a). However, cRNA and vRNA production was significantly reduced in cells infected with Cal NPCA relative to Cal WT (Figure 3b,c). This difference in vRNA production between Cal WT and Cal NPCA increased further by 12 hpi compared to cRNA production, likely due to multiple rounds of genome replication required to produce progeny vRNAs (vRNA to cRNA to vRNA). This reduction of vRNA synthesis also affected mRNA production at 12 hpi (Figure 3a). These data suggest that the efficiency of NP production regulated by GRSF1 association to NP mRNA has a strong impact on viral genome replication and progeny virus production.

### 3.4. Effect of Mutation of GRSF1 Binding Site on Nuclear Export of NP mRNA

**Figure 4:**
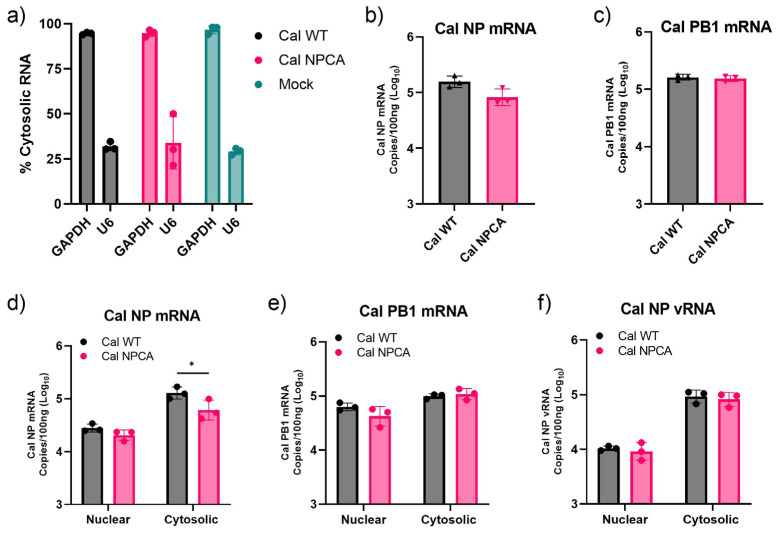
Reduced nuclear export of NP mRNA in NPCA virus-infected cells. A549 cells were infected at an MOI of 3. At 8 hpi, cells were fractionated into nuclear and cytosolic fractions and RNAs were quantitated by qRT-PCR. (**a**) Percentages of GAPDH mRNA and U6 snRNA in cytosolic fraction. (**b**,**c**) NP (**b**) and PB1 (**c**) mRNAs in nuclear and cytosolic fractions were quantified and total mRNAs were calculated. (**d**–**f**) Quantities of NP mRNA (**d**), PB1 mRNA (**e**), and NP vRNA (**f**) in nuclear and cytosolic fractions. All error bars show means plus/minus the standard deviations (n  =  3 biological replicates). Two-way ANOVA followed by Tukey’s multiple comparison test in PRISM (* *p*  <  0.05).

We previously detected reduced nuclear export of NPCA mRNA produced by a Cal polymerase expressed from cDNAs in transfected cells [9]. Here, we tested if the CA mutation also affects the nuclear export of viral mRNAs in infected cells. A549 cells were infected with either Cal WT or Cal NPCA and cell lysates were separated into nuclear and cytosolic fractions at 8 hpi. NP and PB1 mRNAs in each fraction were quantified using qRT-PCR. First, we measured quantities of cytosolic GAPDH mRNA and nuclear U6 snRNA in each fraction and confirmed the efficacy of sample fractionation (Figure 4a). We also measured total NP and PB1 mRNAs, which were similar between Cal WT and Cal NPCA (Figure 4b,c). This agrees with previous data that primary transcription is not affected by the GRSF1 binding site mutation (Figure 3a). Quantification of NP mRNA in each fraction showed that there was a significant reduction of cytosolic NP mRNA in Cal NPCA-infected cells (Figure 4d). In contrast, there was no difference in cytosolic PB1 mRNA between Cal WT- and Cal NPCA-infected cells (Figure 4e). These results indicate that the mutation of the NP gene in the GRSF1 binding site specifically reduces the nuclear export of NP mRNA.

IAV mRNA, cRNA, and vRNA all utilize different pathways for nuclear export [28,29,30]. It is well established that progeny viral ribonucleoprotein complexes (vRNPs) are exported to the cytoplasm via chromosome region maintenance 1 (CRM1) nuclear exporter. Therefore, we tested whether the GRSF1 binding mutation would also affect vRNA nuclear export in infected cells. We found no reduction of cytosolic NP vRNP in Cal NPCA-infected cells (Figure 4f). These results strongly suggest that GRSF1 specifically plays a role in mRNA nuclear export, leading to enhanced translation.

### 3.5. Ribosome Association and Translation of NP CA mRNA

**Figure 5:**
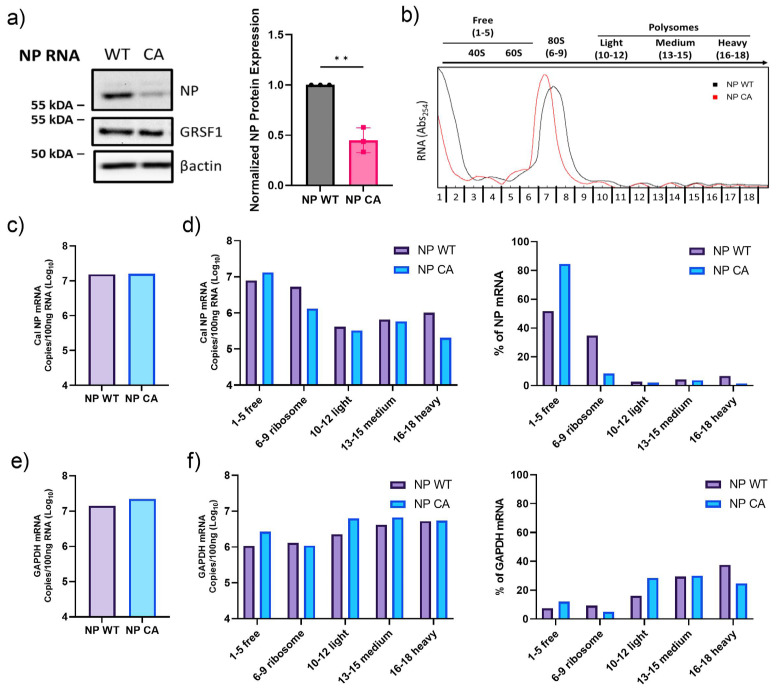
Reduced ribosome association and translation of NPCA mRNA. (**a**) Initially, 293T cells were transfected with NP WT or NPCA mRNA transcribed in vitro. NP protein in transfected cells at 4 h post transfection was determined by Western blot analysis. Relative NP expression was calculated by densitometry analyses and normalized to actin. The data are expressed as NP expressed from NPCA mRNA relative to NP WT mRNA. All error bars show means plus/minus the standard deviations (n  =  3 biological replicates). Unpaired two–tailed t–test in PRISM (** *p*  <  0.01). (**b**) Representative polysome traces from 293T cells transfected with NP WT mRNA (black) or NPCA mRNA (red). (**c**–**f**) NP mRNA and cellular GAPDH mRNA in fractionated cell lysates were quantified by qRT-PCR and grouped into free (fractions 1–5), ribosome (fractions 6–9), light polysomes (fractions 10–12), medium polysomes (13–15), or heavy polysomes (fractions 16–18). (**c**) Total NP mRNA was calculated by summing all fractions. (**d**) NP mRNAs are presented as value per grouped fractions and percentage of total NP mRNA. (**e**) Total GAPDH mRNA was calculated by summing all fractions. (**f**) GAPDH mRNAs are presented as value per grouped fractions and percentage of total GAPDH mRNA (n  =  1 biological replicate).

As described above, we observed that mutation in the GRSF1 binding site in NP mRNA resulted in reduced nuclear export of NP mRNA, production of NP protein, and replication of the viral genome, which caused attenuated virus growth in infected cells (Figure 1, Figure 2, Figure 3 and Figure 4). Reduced NP production could be due to inefficient nuclear export of NP mRNA. However, it is also possible that GRSF1 further contributes to efficient translation of viral mRNA through recruitment of ribosomes. To directly test whether the GRSF1 binding site mutation in NP mRNA affects the translation efficiency of the mRNA, we transcribed capped and polyadenylated WT and CA mRNAs in vitro and transfected each RNA into 293T cells. Cell lysates were collected 4 h after transfection and the NP protein level was analyzed by Western blot. We found that NPCA mRNA was translated significantly less than WT mRNA (Figure 5a), suggesting that the GRSF1 binding site contributes to the enhanced translation of the mRNA.

To further characterize the interaction between the transfected mRNAs and ribosomes, we performed polysome fractionation of transfected cell lysates. Cell lysates transfected with in vitro-transcribed NP WT or NPCA mRNA were applied to ultracentrifugation through sucrose gradients and fractionated. NP WT or NPCA mRNA in each fraction was quantitated to reveal mRNA association with ribosomes and polysomes (Figure 5b). There was no difference in total mRNA between the NP WT and NPCA transfected cells, confirming the same amount of mRNA was initially transfected (Figure 5c). However, we observed differences in the distribution of NP WT and NPCA mRNAs among the fractions (Figure 5d). We detected more NP WT mRNA recovered from ribosome- or polysome-containing fractions compared to NPCA mRNA. Overall, 48% of NP WT mRNA was recovered from ribosome- or polysome-containing fractions compared to 15% of NPCA mRNA (fractions 6–18). We also quantitated GAPDH mRNA to confirm that this difference of NP mRNA association in transfected cells is not due to any global effects of mRNA transfection on translation. The quantity of total GAPDH mRNA was not different between NP WT and NPCA mRNA transfected cells. (Figure 5e). Additionally, the distribution of GAPDH mRNA was not different between the samples (Figure 5f). These results indicate that the GRSF1 binding site plays a significant role in both the nuclear export and translation of IAV NP mRNA.

## 4. Discussion

Emerging IAVs derived from avian or swine IAVs acquired various mutations in viral genes to infect and maintain circulation among human hosts [22,31,32,33]. In the case of the most recent 2009 pH1N1, we found that mutations in the polymerase subunit PA have a strong impact on the IAV human adaptation process [6,9]. Host adaptive mutations in the PA NTD enhanced the translation efficiency of NP mRNA, rather than directly enhancing polymerase activity. This segment-specific activity was due to the increased recruitment of GRSF1 to viral NP mRNAs, which contributed to the efficient nuclear export of NP mRNA and production of NP protein. Here, we directly assessed the impacts of GRSF1 in the life cycle of IAV using a rescued mutant virus. We found that a mutation in the GRSF1 binding site in the NP 5’ UTR significantly reduced the nuclear export of NP mRNA, resulting in the reduced expression of NP protein. The reduced NP expression led to restricted genome replication and reduced overall viral growth. GRSF1 association with NP mRNA to enhance the translation efficiency appears to be a key for viral growth. Underscoring the importance of GRSF1, pH1N1 acquired an additional PA NTD mutation, V100I, during seasonal circulation. The additional V100I mutation further enhanced the translation efficiency of NP mRNA [9]. This suggests that GRSF1-mediated enhancement of NP mRNA translation is an effective strategy of pH1N1 host adaptation during seasonal circulation, allowing rapid virus genome replication and progeny virion release.

IAV has been recognized to utilize various host factors for mRNA export from the nucleus. Cellular mRNAs are typically exported by a complex of proteins that associates with the mRNA during the transcription and processing steps [34]. Unlike cellular mRNAs, IAV mRNAs are transcribed by the viral polymerase, many of which, including NP, are not spliced. The mechanism of nuclear export of IAV mRNAs has not been fully elucidated [35,36]. Previous research has revealed that IAV mRNA export pathways are largely segment- and cell type-specific. Studies in 293T cells have shown that the nuclear export of NP mRNA is not affected by NXF1 depletion, unlike HA, M1, M2, or NS1 mRNAs [37]. In contrast, studies in A549 cells using dominant-negative NXF1 have shown that NP mRNA nuclear export is NXF1-dependent [28]. Both studies showed that the nuclear export of NP mRNA is not reliant on CRM1 export pathways [28,37]. Additionally, work in A549 cells found that polymerase PA, PB1, and PB2 mRNAs did not use either NXF1 or CRM1, suggesting the use of an atypical export pathway [28]. Utilizing different pathways for different segments could be an approach to regulate the levels of viral protein expression since the precise adjustment of protein levels is required for efficient virus growth.

It is noteworthy that IAV utilizes host GRSF1 to accelerate NP mRNA translocation and translation. So far, limited data are available on host RNA binding proteins (RBPs) associated with viral mRNAs. However, a recent study using protein–RNA cross-linking and RNA interactome analysis identified 51 host proteins associated with IAV NP mRNA [38]. The list of identified proteins includes GRSF1 and other members of hnRNPs, as well as splicing factors known to be involved in the life cycle of IAV [36]. Although the association of the identified hnRNPs or splicing factors to mRNA was not NP mRNA-specific, their data indicate the presence of various RBPs bound to viral mRNAs in the life cycle of IAV. Interestingly, identified RBPs also interacted with the viral polymerase complex, like we had observed in enhanced GRSF1 association by mutations in the PA subunit. These findings suggest that IAV polymerase can regulate the association of RBPs to viral mRNAs to regulate their protein expression.

Enhanced association of GRSF1 to specific viral mRNAs, such as NP and NS1, could be an important factor for human host adaptation and sustaining viral transmission as population immunity increases during continuous circulation. It is not known whether avian IAVs utilize GRSF1 during infection in avian hosts. Because the GRSF1 binding sequence is also conserved in the NP and NS genes of avian IAVs, it is likely that avian IAVs also utilize the avian GRSF1 to enhance the expression of the NP or NS1 proteins [9]. GRSF1 contains an N-terminal alanine-rich domain, three RNA binding domains (RBDs), and an acidic regulatory domain [39,40,41]. The overall homology of chicken GRSF1 (NCBI: XP_015131904.2) to human GRSF1 is not high (43%). While the sequence of three RBDs is relatively well conserved (67%, 60%, and 60% respectively), the acid domain that is thought to be responsible for the regulation of protein–protein interactions shares only 22% homology. Therefore, it is possible that IAV polymerase requires mutations at key residues in PA to efficiently recruit and attach human GRSF1 to viral mRNAs. Our data suggest that IAVs utilize GRSF1 to enhance NP expression to accelerate genome replication and viral growth. Rapid virus growth is likely to be a key factor to sustain viral transmission as population immunity increases during seasonal circulation.

## Data Availability

All study data are included in this article.
